# Psychological characteristics associated with the brain volume of patients with fibromyalgia

**DOI:** 10.1186/s13030-023-00293-2

**Published:** 2023-10-24

**Authors:** Satoshi Izuno, Kazufumi Yoshihara, Masako Hosoi, Sanami Eto, Naoki Hirabayashi, Tae Todani, Motoharu Gondo, Chie Hayaki, Kozo Anno, Akio Hiwatashi, Nobuyuki Sudo

**Affiliations:** 1 Department of Psychosomatic Medicine, Graduate School of Medical Sciences, Kyushu University, 3-1-1 Maidashi, Higashiku, Fukuoka, Fukuoka 812-8582 Japan; 2 Department of Psychosomatic Medicine, Kyushu University Hospital, Fukuoka, Japan; 3 Multidisciplinary Pain Center, Kyushu University Hospital, Fukuoka, Japan; 4 Kyushu University Ito Clinic, Fukuoka, Japan; 5 Department of Psychosomatic Medicine, Kyushu Central Hospital of the Mutual Aid Association of Public School Teachers, Fukuoka, Japan; 6 Department of Radiology, Graduate School of Medical Sciences, Nagoya City University, Nagoya, Japan

**Keywords:** Fibromyalgia, Voxel-based morphometry, Gray matter, White matter, Pain catastrophizing

## Abstract

Fibromyalgia (FM) is a disease characterized by chronic widespread pain concomitant with psychiatric symptoms such as anxiety and depression. It has been reported that FM patients engage in pain catastrophizing. In this study, we investigated characteristics of the brain volume of female FM patients and the association between psychological indices and brain volume. Thirty-nine female FM patients and 25 female healthy controls (HCs) were recruited for the study, and five FM patients were excluded due to white matter lesions. The following analyses were performed: (1) T1-weighted MRI were acquired for 34 FM patients (age 41.6 ± 7.4) and 25 HCs (age 39.5 ± 7.4). SPM12 was used to compare their gray and white matter volumes. (2) Data from anxiety and depression questionnaires (State-Trait Anxiety Inventory and Hospital Anxiety and Depression Scale), the Pain Catastrophizing Scale (subscales rumination, helplessness, magnification), and MRI were acquired for 34 FM patients (age 41.6 ± 7.4). Correlation analysis was done of the psychological indices and brain volume. We found that (1) The white matter volume of the temporal pole was larger in the FM patient group than in the HC group. (2) Correlation analysis of the psychological indices and gray matter volume showed a negative correlation between trait anxiety and the amygdala. For the white matter volume, positive correlations were found between depression and the brainstem and between magnification and the postcentral gyrus. Changes in the brain volume of female FM patients may be related to anxiety, depression, and pain catastrophizing.

## Introduction

Fibromyalgia (FM) is a disease characterized by chronic widespread pain concomitant with various physical and psychiatric symptoms such as fatigue, sleep disturbances, and depression. It has been reported that the prevalence of FM in the general population is 0.2–6.6% [1] and that it is common among middle-aged women [2]. Because FM patients have a reduced quality of life and significant limitations in social activities, there is an urgent need to understand the pathogenesis. Data on genetic, immunological, and psychosocial factors have been accumulated: however, at present there is no consistent pathological model of FM.

The pathogenesis of fibromyalgia has been found to be closely related to its psychological factors. Psychiatric symptoms such as depression and anxiety have been found to influence changes in pain thresholds [3, 4]. Regarding the psychiatric comorbidities of FM patients, it has been reported that the prevalence of psychiatric disorders is significantly higher in FM patients compared to healthy controls (HCs) and that depression is found in 20–80% and anxiety disorders in 20–60% [5, 6]. Pain catastrophizing is a common psychological factor of chronic pain [7]. Catastrophizing is a concept proposed by Albert Ellis and defined as an irrationally negative forecast of future events [8]. Pain catastrophizing refers to a maladaptive cognitive style in which the person overestimates present and future pain-related disability and is trapped in that belief [8]. In the treatment of FM, the therapeutic prognosis is defined by depression, anxiety, and pain catastrophizing, and pain becomes treatment-resistant when severe [9].

Recent technological advances in medical imaging equipment have resulted in magnetic resonance imaging (MRI) being able to be used to elucidate the neural basis of pain and to capture structural changes in the brain of patients with chronic pain disorders. Brain morphological studies of FM have noted changes in several brain regions, including the cingulate cortex, medial prefrontal cortex, parahippocampal gyrus, postcentral gyrus, angular gyrus, and cerebellum [10–13], but the data are inconsistent and debatable. As mentioned as a limitation of these meta-analyses, this may be due to the lack of subtype and meta-regression analyses to control for relevant factors that may influence brain structural changes in FM patients, such as disease severity, disease duration, comorbidity, and emotional state. A study examining the correlation between the gray matter brain volumes and clinical symptoms of FM patients found a positive correlation between pain severity and the superior temporal gyrus, a positive correlation between trait anxiety and the medial orbitofrontal cortex, a negative correlation between psychological distress and the medial orbitofrontal cortex, and a positive correlation between psychological distress and the cerebellum [14]. Another research group has reported that pain threshold correlates negatively with gray matter volume in the middle cingulate cortex (MCC), posterior cingulate cortex (PCC), and cerebellum and that the chronicity of pain correlates negatively with gray matter volume in the insular cortex and rolandic operculum [15].

Regarding white matter abnormalities, most studies have examined white matter microstructure using diffusion tensor imaging; in FM patients, changes have been noted in multiple white matter tracts, including the corpus callosum, internal capsule, and corona radiata [15–17], but are inconsistent and have limitations as well. Among the above, studies examining associations with clinical symptoms have noted associations with pain intensity and psychiatric symptoms such as depression and anxiety, so it would be useful to examine the pathogenesis of FM based on changes in white matter structures.

However, these previous studies have focused on changes in gray matter volume or white matter microstructure, with few studies examining changes in white matter volume. In addition, few neuroimaging studies have included pain catastrophizing, which is one of the psychological characteristics of FM patients. In this study, we investigated the characteristics of the brain volume of female FM patients, including white matter volume, and the association between brain volume and psychological indices, including pain catastrophizing, focusing on several brain regions involved in emotion. The cingulate cortex is involved in emotional processing, memory, and learning and serves as a hub for various brain regions [18]. The cerebellum has traditionally been thought to be associated with motor control, but recently it has been revealed that it is also involved in cognition, emotion, and personality traits [19]. In particular, Crus1 of the cerebellum is thought to be responsible for social cognition, which involves inferring intentions and mental states from the behavior of others [20]. These regions have been noted to have altered brain volumes in meta-analyses of FM brain morphology studies [10, 11]. These previous studies reported changes in gray matter; however, we thought it would be useful to examine white matter changes in that region because gray matter affects the corresponding white matter in healthy subjects [21] and patients with neurological and psychiatric disorders [22, 23], although not with fibromyalgia. Other important regions for emotion and emotional processing are the temporal pole (TP) [24] and the amygdala [25]. The uncinate fasciculus, a white matter tract that connects the rostral temporal regions such as the TP and amygdala to the frontal lobe and is involved in memory and social-emotional processing, has been reported to be abnormal in psychiatric disorders [26, 27]. Therefore, we considered it highly likely that abnormal gray and white matter volumes in those brain regions would also be found in fibromyalgia, which is often associated with psychiatric symptoms. In addition, when focusing on pain catastrophizing, we hypothesized that the brain regions associated with pain [28] (insula [29], postcentral gyrus [30], and brainstem [31]) would be abnormal, and examined changes in their gray matter and white matter volumes.

## Methods

### Participants

The data of 39 right-handed Japanese female FM patients who visited Kyushu University Hospital between 2014 and 2020 was available for study. All had had MRI taken and psychological indices administered during that period. The diagnosis of FM was made by an experienced psychosomatic physician following the American College of Rheumatology (ACR) 2010 diagnostic criteria [32]. According to these criteria, fibromyalgia is diagnosed when the following three criteria are met: (1) widespread pain index (WPI) ≧ 7 and symptom severity (SS) ≧ 5, including fatigue, waking unrefreshed, and cognitive symptoms, or WPI 3–6 and SS ≧ 9, (2) persistence of clinical symptoms for at least three months, and (3) absence of other conditions that would explain the chronic pain. HCs were recruited from the local population by advertisement. They were 25 right-handed Japanese women without any history of chronic pain or psychiatric disorder. All participants gave written informed consent before the study. The study was approved by the ethics committee of Kyushu University Hospital (29–543).

Of the 39 patients recruited to the FM group, the data of 34 were available for the analysis after excluding five with white matter lesions that might prevent accurate segmentation. Because of the apparent effects of sex on brain structure [33], this study included only female patients to exclude its confounding. Age was also included in the covariates to exclude the effect of age on brain structure [34]. As a reference, 25 HCs and 25 age-matched FM patients were selected from the 34 participant FM group to be included in the analysis of the comparisons between the two groups.

### Psychological assessment

Psychological indices were given as self-administered questionnaires. The Japanese version of the Center for Epidemiologic Studies Depression Scale (CES-D) was used to assess depression [35, 36] and the Japanese version of the State-Trait Anxiety Inventory (STAI) to assess anxiety [37, 38]. The STAI assesses two types of anxiety: state anxiety (anxiety currently felt) and trait anxiety (tendency to feel anxiety). The Japanese version of the Hospital Anxiety and Depression Scale (HADS) was used to assess psychological distress [39, 40]. The HADS consists of two subscales: depression and anxiety. The Japanese version of the Pain Catastrophizing Scale (PCS) was used to assess pain catastrophizing [41, 42]. The PCS consists of three subscales: rumination, helplessness, and magnification. The reliability and validity of the Japanese versions of the psychological indices used have been verified and reported (CES-D [36], STAI [37], HADS [39], and PCS [41]).

Only CES-D and STAI were administered to the HC group: of the 25 HCs, three had missing measurement data on CES-D as did three on STAI, so their data were excluded from the analysis.

### MRI data acquisition

MRI data were acquired on a 3.0-Tesla MRI scanner (Achieva TX, Philips Healthcare, Best, The Netherlands). T1-weighted 3D images were collected using a magnetization prepared rapid gradient echo (MP-RAGE) sequence with the following parameters: repetition time = 7.9 ms, echo time = 3.7 ms, flip angle = 9°, field of view = 256 × 256 mm, acquisition matrix = 256 × 256, number of slices = 200 slice thickness = 1.0 mm.

### MRI data analysis

The T1-weighted images were manually checked for morphological abnormalities, brain lesions and artifacts. White matter lesions were observed in five FM patients, so the data of these five were excluded from the analysis. The other T1-weighted images were aligned to the anterior-posterior commissures line to correct for head tilt. Data processing and analysis were done using Statistical Parametric Mapping 12 (SPM12 http://www.fil.ion.ucl.ac.uk/spm). Voxel-based morphometry (VBM) was performed using SPM12 running in MATLAB R2013b (MathWorks, Inc., Natick, MA, USA). In brief, we performed segmentation of gray matter, white matter, and cerebrospinal fluid and spatial normalization onto the Montreal Neurological Institute (MNI) space using the Diffeomorphic Anatomical Registration using Exponentiated Lie Algebra (DARTEL) algorithm [43]. The gray matter/white matter maps were then smoothed with an 8 mm full width at half maximum isotropic gaussian kernel.

The preprocessed data of all subjects were entered into a full factorial analysis (analysis of covariance) using the SPM12 general linear model. Age and total brain volume were included as covariates in the model. In the comparison of FM patients and HCs, we examined differences in regional gray matter/white matter volumes of the whole brain or regions of interest (ROIs). In the correlation analysis, we examined the correlation between each psychological index and its subscales and the regional gray matter/white matter volumes of the whole brain or ROIs of the FM patients. Findings were considered significant at a cluster level of *p* < 0.05 corrected for multiple comparisons (family-wise error FWE). As ROIs, we used the cingulate cortex, cerebellum, amygdala, TP, insula, postcentral gyrus, and brainstem. We defined all ROIs using the WFU_PickAtlas that incorporates the automatic anatomical labeling (AAL) atlas and assessed left and right ROIs combined. Four ROIs (cingulate cortex, cerebellum, TP, amygdala) were used for the results of the between group comparison, two (TP, amygdala) for the results of the correlation analysis with trait anxiety, and three (insula, postcentral gyrus, brainstem) for the results of the correlation analysis with catastrophizing. We performed a multiple comparison correction for the number of ROIs using the Bonferroni method.

### Statistical analysis

The data of 34 FM patients and 25 HCs were available for between group comparisons with age and total brain volume as covariates, and the data of 34 FM patients were available for the correlation analysis of the psychological indices and brain volume with age as covariates. In addition, to exclude the effect of age on brain structure more strictly, we compared the gray and white matter volumes of 25 FM patients and 25 age matched HCs, with total brain volume as covariates.

Comparison of the psychological indices was performed using the data of the CES-D and STAI. Normality was assessed by the Shapiro-Wilk normality test. In the case of normal distribution, statistical differences between groups were analyzed by Student’s t-test for normal distribution and by the Kruskal-Wallis test for non-normal distribution.

## Results

### Between-group differences in psychological indices

The psychological indices of the 25 HCs, the 34 FM patients, and the 25 age matched FM patients are shown in Table 1. The between group comparison showed that the scores on the CES-D and STAI were significantly higher in the FM group (each *p* < 0.0001).

**Table 1:** Participant age and psychological characteristics

	HC (n = 25) *	FM (n = 34)	FM (n = 25)	*p* value	
				25 HC vs. 34 FM	25 HC vs. 25 FM
Age	39.5 ± 7.4	41.6 ± 7.4	39.7 ± 6.7	0.29	0.92
CES-D	7.3 ± 6.1	30.2 ± 12.6	31.8 ± 11.8	< 0.001	< 0.001
STAI					
State	37.0 ± 12.1	55.7 ± 10.8	56.5 ± 10.8	< 0.001	< 0.001
Trait	36.9 ± 10.5	59.6 ± 12.8	60.4 ± 12.5	< 0.001	< 0.001
HADS					
Depression		11.0 ± 4.4	11.4 ± 4.1		
Anxiety		10.5 ± 4.2	10.5 ± 3.7		
PCS					
total		32.7 ± 12.0	33.2 ± 11.4		
Rumination		14.2 ± 4.7	14.5 ± 4.5		
Helplessness		11.8 ± 5.0	11.9 ± 4.2		
Magnification		6.7 ± 3.6	6.8 ± 3.7		

*CES-D (n = 22), STAI (n = 22)

### Between-group differences in brain volume

**Fig. 1:**
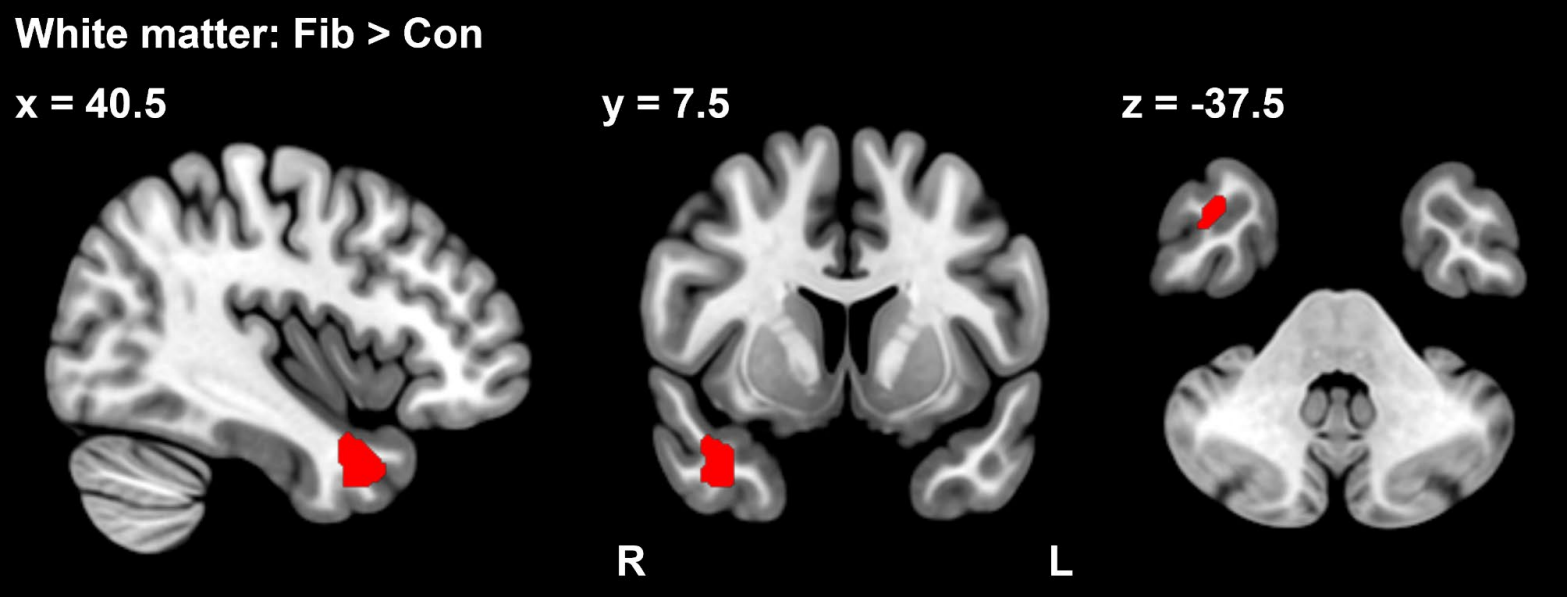
Between-group differences in white matter volume. Fibromyalgia patients had a significant increase of white matter volume in the right temporal pole (TP) (red area), compared to healthy controls (*p*_FWE_ < 0.05, ROI: TP).

No significant difference in the gray or white matter volume of the 34 FM patients compared to the 25 HCs was found in whole-brain analysis. ROI analysis showed an increased white matter volume in the TP of the FM group (*p*_FWE_ < 0.05, ROI: TP) (Table 2) (Fig. 1) In the comparison of the 25 FM patients and the 25 age matched HCs, ROI analysis showed an increased white matter volume in the TP of the FM group (*p*_FWE_ < 0.05, ROI: TP) (Table 2). However, the result was not significant after correcting for the number of ROIs.

**Table 2:** Regions of greater white matter volume for fibromyalgia patients than for healthy controls

Brain region	MNI coordinates			cluster size	peak t value	*p* value (FWE)
	x	y	z			
34 FM > 25 HC						
R Temporal Pole	40.5	7.5	-37.5	198	5.16	0.021
25 FM > 25 HC						
R Temporal Pole	40.5	7.5	-37.5	92	4.57	0.043

### Correlation analysis for the whole brain

**Fig. 2:**
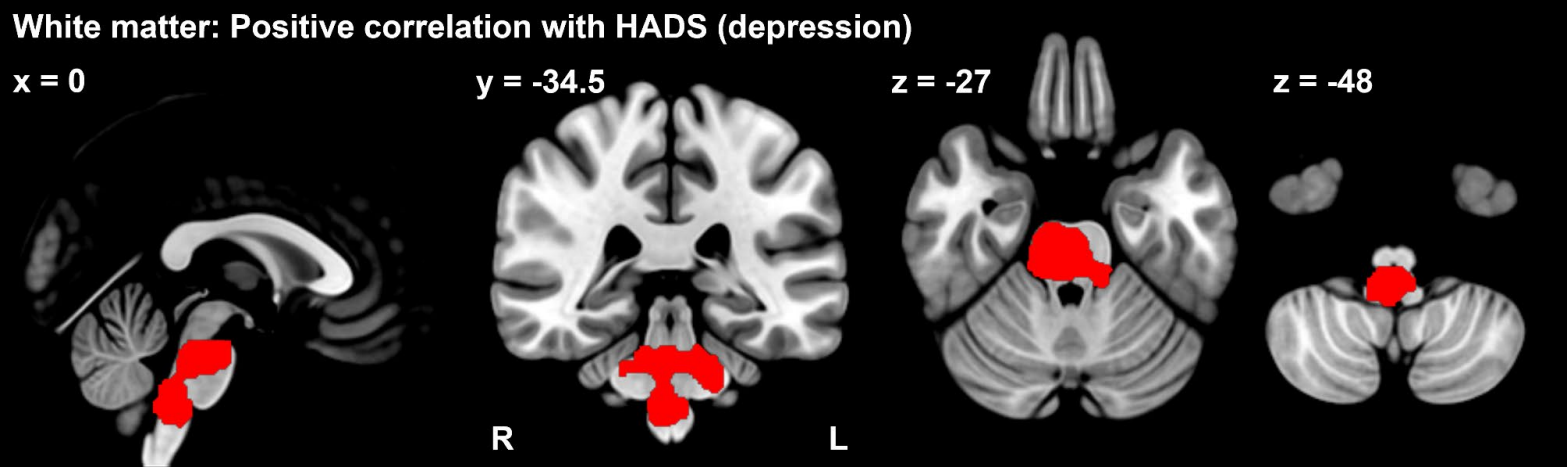
Correlation analysis results for the white matter volume of the whole brain. Fibromyalgia patients had a significant, positive correlation between white matter volume in the brain stem (red area) and the HADS (depression) (*p*_FWE_ < 0.05, whole brain)

Whole-brain correlation analysis with the psychological indices showed a positive correlation between depression on the HADS and white matter volume in brain regions ranging from the pons to the medulla oblongata (*p*_FWE_ < 0.05) (Table 3) (Fig. 2).

**Table 3:** Relation of regional white matter volume with the psychological characteristics of fibromyalgia patients

Brain region	MNI coordinates			cluster size	peak t value	*p* value (FWE)
	x	y	z			
Positive correlation with HADS (Depression)						
Medulla ~ Pons	0	-34.5	-48	4251	5.27	< 0.001
Positive correlation with PCS (Magnification)						
R Postcentral gyrus	42	-28.5	51	237	5.33	0.032

### Correlation analysis with ROIs

**Fig. 3:**
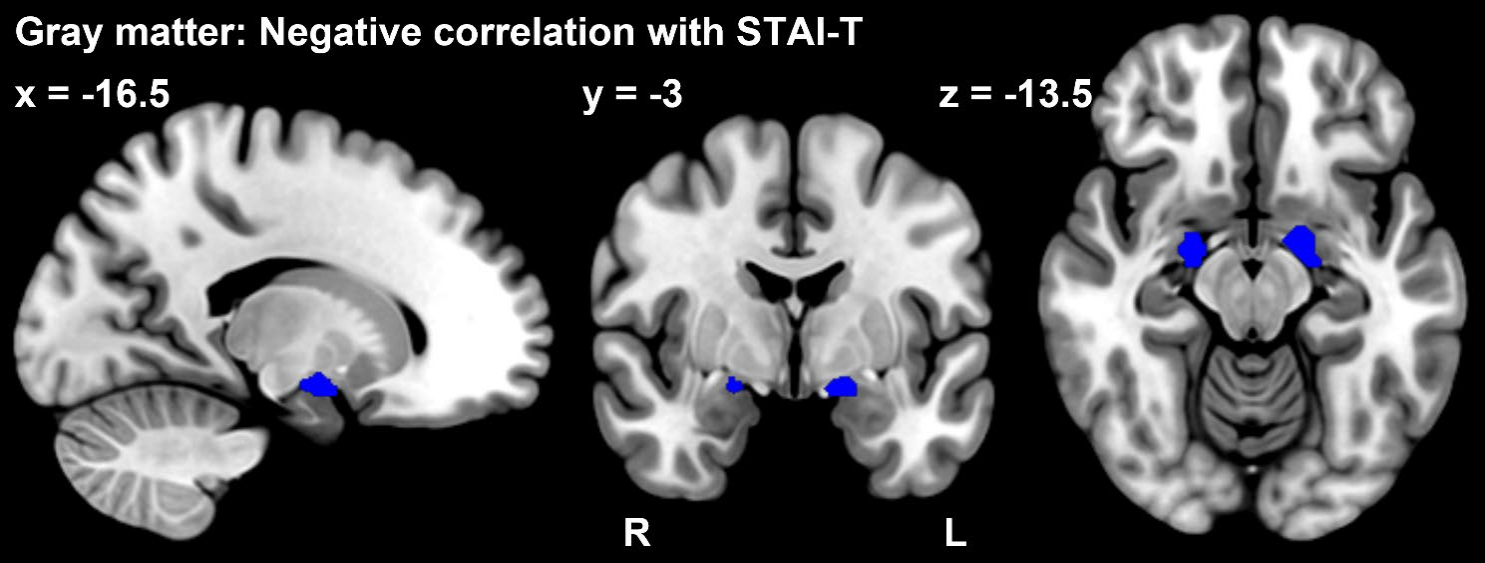
Correlation analysis results for the gray matter volume in ROIs. Fibromyalgia patients had a significant, negative correlation between the gray matter volume of the amygdala (blue area) and the STAI (trait anxiety) (*p*_FWE_ < 0.05, ROI: amygdala)

**Fig. 4:**
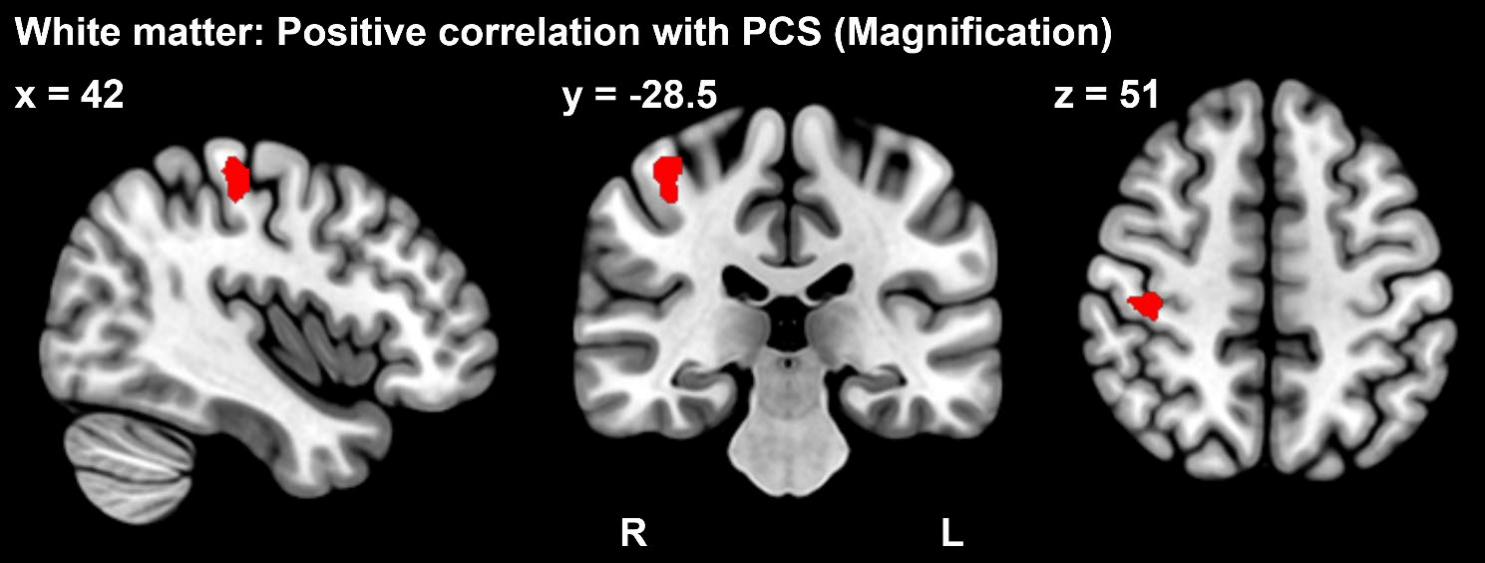
Correlation analysis results for the white matter volume in ROIs. Fibromyalgia patients had a significant, positive correlation between white matter volume in the postcentral gyrus (red area) and PCS (magnification) (*p*_FWE_ < 0.05, ROI: postcentral gyrus)

Correlation analysis with psychological indices using ROIs showed a negative correlation between trait anxiety and gray matter volume in the bilateral amygdala (*p*_FWE_ < 0.05, ROI: amygdala) (Table 4.) (Fig. 3.). The result was significant after correcting for the number of ROIs. We also found positive correlations between magnification of the PCS and the white matter volume of the postcentral gyrus (*p*_FWE_ < 0.05, ROI: postcentral gyrus) (Table 3) (Fig. 4). However, the result was not significant after correcting for the number of ROIs.

**Table 4:** Relation of regional gray matter volume with the psychological characteristics of fibromyalgia patients

Brain region	MNI coordinates			cluster size	peak t value	*p* value (FWE)
	x	y	z			
Negative correlation with STAI-T						
L Amygdala	-16.5	-3	-13.5	55	4.23	0.019
R Amygdala	21	-6	-13.5	36	3.92	0.024

## Discussion

This study used VBM to investigate differences in the brain volume of FM patients and HCs and the association between psychological traits and the brain volume of FM patients. By focusing on changes in white matter volume as well as gray matter, we were able by ROI analysis (uncorrected) to report for the first time that FM patients had greater white matter volume in the TP than did HCs. The correlation analysis of psychological indices and the gray matter volume of the FM group showed a negative correlation between trait anxiety and the amygdala, also by ROI analysis (corrected). The correlation analysis of psychological indices and white matter volume showed positive correlations between depression and the brainstem by whole brain analysis, and between magnification and the postcentral gyrus by ROI analysis (uncorrected). In particular, an association between pain catastrophizing, a psychological feature of FM patients, and brain volume was revealed for the first time. However, the impact of the results of the between group comparison and the correlation with magnification is unclear because the results were not significant after correcting for the number of ROIs.

We found that the FM group had a greater white matter increase in the right TP when compared to the HC group, which might reflect cognitive distortions specific to FM patients. The TP is a phylogenetically recent cell structure and unique to primates, which suggests that it is responsible for high level cognitive function [24]. Recent studies have reported that it is responsible for several different functions, including high-order visual perception, such as faces and complex objects [44], verbal semantic memory [45], autobiographical memory [46], and social cognition [47], such as emotional processing and empathy. Previous studies on white matter volume have reported increased volume in the right TP, with higher automatic thoughts scores in healthy subjects [48]. Automatic thoughts are thoughts or images that come to mind momentarily when an event occurs. If they are overly negative, they can lead to depression [49] and emotional and behavioral problems [50]. An association between automatic thoughts and pain-related disorders has also been noted, with reports that patients with irritable bowel syndrome [51] and tension-type headache [52] have higher automatic thoughts scores than HCs. Moreover, Chronic pain patients with depression have been found to have significantly more negative automatic thoughts than patients without depression or healthy controls [53]. Functional MRI studies have shown that the higher the fear of pain, the greater the TP activation during pain anticipation in healthy subjects [54], and that TP activation is greater during pain stimulation in patients with migraine than HCs [55]. White matter volume change in the TP might be associated with negative automatic thoughts or chronic pain processing, as shown by the FM group in this study also scoring higher in depression.

On the other hand, there was no between group difference in gray matter volume, which is inconsistent with previous studies, but this may be due to the heterogeneous population of the FM group. Previous studies comparing the brain volume of FM patients and HCs have noted changes in various brain regions, with inconsistent results [14, 34, 56–61]. Most of these studies used the ACR 1990 diagnostic criteria [62], which diagnoses FM by focusing on the number of tender points, as inclusion criteria for their FM group. Our study used the ACR 2010 diagnostic criteria. These criteria include clinical symptoms other than pain and have increased specificity; however, they allow fibromyalgia to be diagnosed only based on clinical symptoms, thus the possibility remains that the FM group is a heterogeneous population. This may have influenced the inconsistent results for the brain volume characteristics of the FM patients.

Our correlation analysis between gray matter volume and the psychological indices of FM patients showed that the gray matter volume in the amygdala decreased with higher trait anxiety. The amygdala plays an important role in brain circuits related to anxiety and fear. The amygdala receives input from the cortex and thalamus, integrates and processes this information, and projects it to several regions, including the periaqueductal gray matter basal nucleus of the striatum, hypothalamus, and dorsal vagal complex, to bring anxiety- and fear-related behavior such as avoidance [63]. A meta-analysis examining amygdala size in patients with chronic pain found significantly higher emotional stress and significantly smaller amygdala volume compared to healthy controls, consistent with the present results [64]. Previous studies examining correlates of trait anxiety have reported an association between trait anxiety and both increased and decreased amygdala volume in healthy subjects [65, 66], whereas, for pathological conditions such as panic disorder, it has been reported that decreased amygdala volume is associated with higher trait anxiety [67]. The negative correlation between trait anxiety and gray matter volume in the amygdala found in this study may reflect pathological anxiety in FM patients.

Our correlation analysis of white matter volume and the psychological indices of FM patients showed that the white matter volume in brain regions ranging from the pons to the medulla oblongata and cerebellum increased with higher depression. A previous study reported that patients with major depressive disorders (MDD) have a larger white matter volume, ranging from the midbrain to the upper pons, compared to HCs [68], which overlaps with the brain regions where volume changes were observed in this study. This region contains the raphe nuclei, a cell population of serotonergic neurons that is related to depression [69]. In addition, a study using diffusion tensor imaging analysis found that patients with MDDs have greater mean diffusivity values in the pons, indicating the possible presence of demyelination and inflammation [70]. A study using resting-state functional brain imaging analysis found that patients with MDDs have increased functional connectivity between the medulla oblongata and inferior parietal lobes [71]. There is growing evidence for a link between depression and the brainstem. The white matter volume change in the brainstem shown in this study may reflect change due to the depression of FM patients, similar to the changes observed for MDDs. Moreover, the brain region contains the locus coeruleus (LC), where noradrenergic neurons are assembled. Noradrenaline is associated not only with depression but also with pain processing, and the LC plays an important role in the descending pain inhibitory system [31]. It has been shown that as the pain becomes chronic, the activity of the LC gradually decreases and the descending pain inhibitory system is impaired [72], which may be reflected by the change in pontine volume.

In addition, our correlation analysis of white matter volume and the psychological indices of FM patients showed that the white matter volume in the right postcentral gyrus increased with stronger pain magnification. The postcentral gyrus corresponds to the primary somatosensory cortex and is closely associated with chronic pain. It is hypothesized that peripheral nerve and tissue injury causes synaptic remodeling of the postcentral gyrus via astrocytes, inducing local hyperactivity of the postcentral gyrus in response to peripheral stimuli [30]. Previous studies using diffusion tensor imaging have reported abnormalities of the postcentral gyrus of patients with FM [73] and other pain-related disorders (irritable bowel syndrome [74], and neuropathic pain [75]). This indicates that white matter volume in the postcentral gyrus might be associated with the pain magnification of pain-related patients.

This study has several limitations. First, although it is relatively large compared to other studies that have examined the brain volume of FM patients, the sample size is still considered to be moderate, thus future study with larger sample size is needed. In addition, although the sample size for this study was selected according to previous publications, power analysis was not performed. Second, as a cross-sectional study, it is not clear if the changes in brain volume revealed in this study are responsible for the psychological symptoms of FM. Longitudinal studies that examine brain volume and psychological symptoms before and after treatment intervention are warranted. Third, the HCs did not complete the HADS and PCS indices. Although not having this data may have limited our discussion, the mean scores of the FM patients were higher than those of the healthy subjects reported in previous studies [39, 41]. Fourth, as noted in the discussion, the results of between group comparison and the correlation with magnification were not significant after performing multiple comparison correction for the number of ROIs. On the other hand, the result of the correlation with trait anxiety remained significant.

In summary, FM patients were found to have a larger white matter volume in the TP than did HCs: the change may be associated with negative automatic thoughts. In addition, the trait anxiety, depression, and magnification of pain catastrophizing of FM patients may be associated with the gray matter volume in the amygdala, white matter volume in the pons and medulla oblongata, and white matter volume in the right postcentral gyrus, respectively.

## Data Availability

The data that support the findings of this study are available on request from the corresponding author KY. The data are not publicly available due to their containing information that could compromise the privacy of research participants.

## List of Abbreviations

AAL: Automatic anatomical labeling
ACR: American College of Rheumatology
CES-D: Center for Epidemiologic Studies Depression Scale
DARTEL: Diffeomorphic Anatomical Registration using Exponentiated Lie Algebra
FM: Fibromyalgia
FWE: Family-wise error
HADS: Hospital Anxiety and Depression Scale
HC: Healthy control
LC: Locus coeruleus
MDD: Major depressive disorders
MNI: Montreal Neurological Institute
MP-RAGE: Magnetization prepared rapid gradient echo
MRI: Magnetic resonance imaging
PCS: Pain Catastrophizing Scale
ROI: Regions of interest
SPM12: Statistical Parametric Mapping 12
STAI: the State-Trait Anxiety Inventory
TP: Temporal pole

## References

[1] Marques AP, Santo A. S do E, Berssaneti AA, Matsutani LA, Yuan SLK. Prevalence of fibromyalgia: literature review update. Rev Bras Reumatol. 2017;57:356–63. 10.1016/j.rbre.2017.01.005. 10.1016/j.rbre.2017.01.005 28743363

[2] Heidari F, Afshari M, Moosazadeh M. Prevalence of fibromyalgia in general population and patients, a systematic review and meta-analysis. Rheumatol Int. 2017;37:1527–39. 10.1007/s00296-017-3725-2. 10.1007/s00296-017-3725-2 28447207

[3] Michaelides A, Zis P. Depression, anxiety and acute pain: links and management challenges. Postgrad Med. 2019;131:438–44. 10.1080/00325481.2019.1663705. 10.1080/00325481.2019.1663705 31482756

[4] Kim DJ, Mirmina J, Narine S, Wachtel J, Carbajal JM, Fox H et al. Altered physical pain processing in different psychiatric conditions. Neurosci Biobehav Rev. 2022;133. 10.1016/j.neubiorev.2021.12.033. 10.1016/j.neubiorev.2021.12.033 34952034

[5] Arnold LM (2008). Management of fibromyalgia and comorbid psychiatric disorders. J Clin Psychiatry.

[6] Fietta P, Fietta P, Manganelli P. Fibromyalgia and psychiatric disorders. Acta Biomed. 2007;78:88–95. Available from: http://www.ncbi.nlm.nih.gov/pubmed/17933276. 17933276

[7] Edwards RR, Dworkin RH, Sullivan MD, Turk DC, Wasan AD. The Role of Psychosocial Processes in the Development and Maintenance of Chronic Pain. J Pain. 2016;17:T70–92. 10.1016/j.jpain.2016.01.001. 10.1016/j.jpain.2016.01.001 PMC5012303 27586832

[8] Quartana PJ, Campbell CM, Edwards RR. Pain catastrophizing: a critical review. Expert Rev Neurother. 2009;9:745–58. 10.1586/ern.09.34. 10.1586/ERN.09.34 PMC2696024 19402782

[9] Giesecke T, Williams DA, Harris RE, Cupps TR, Tian X, Tian TX (2003). Subgrouping of Fibromyalgia patients on the basis of pressure-Pain thresholds and psychological factors. Arthritis Rheum.

[10] Lin C, Lee S, Weng H. Gray Matter Atrophy within the Default Mode Network of Fibromyalgia: A Meta-Analysis of Voxel-Based Morphometry Studies. Biomed Res Int. 2016;2016:1–9. 10.1155/2016/7296125. 10.1155/2016/7296125 PMC5220433 28105430

[11] Shi H, Yuan C, Dai Z, Ma H, Sheng L. Gray matter abnormalities associated with fibromyalgia: A meta-analysis of voxel-based morphometric studies. Semin Arthritis Rheum. 2016;46:330–7. 10.1016/j.semarthrit.2016.06.002. 10.1016/j.semarthrit.2016.06.002 27989500

[12] Dehghan M, Schmidt-Wilcke T, Pfleiderer B, Eickhoff SB, Petzke F, Harris RE (2016). Coordinate-based (ALE) meta-analysis of brain activation in patients with fibromyalgia. Hum Brain Mapp.

[13] Xin M, Qu Y, Peng X, Zhu D, Cheng S. A systematic review and meta-analysis of voxel-based morphometric studies of fibromyalgia. Front Neurosci. 2023;17. 10.3389/fnins.2023.1164145. 10.3389/fnins.2023.1164145 PMC10203234 37229427

[14] Diaz-piedra C, Guzman MA, Buela-casal G, Catena A. The impact of fibromyalgia symptoms on brain morphometry. Brain Imaging Behav. 2016;1184–97. 10.1007/s11682-015-9485-2. 10.1007/s11682-015-9485-2 26615599

[15] Mosch B, Hagena V, Herpertz S, Diers M. Brain morphometric changes in fibromyalgia and the impact of psychometric and clinical factors: a volumetric and diffusion-tensor imaging study. Arthritis Res Ther. 2023;25:81. 10.1186/s13075-023-03064-0. 10.1186/s13075-023-03064-0 PMC10197341 37208755

[16] Kim DJ, Lim M, Kim JS, Son KM, Kim HA, Chung CK. Altered white matter integrity in the corpus callosum in fibromyalgia patients identified by tract-based spatial statistical analysis. Arthritis Rheumatol. 2014;66:3190–9. 10.1002/art.38771. 10.1002/art.38771 25225152

[17] Tu Y, Wang J, Xiong F, Gao F (2022). Disrupted White Matter microstructure in patients with Fibromyalgia owing predominantly to psychological factors: a diffusion Tensor Imaging Study. Pain Physician.

[18] Rolls ET. The cingulate cortex and limbic systems for action, emotion, and memory. Handb Clin Neurol. 2019;166:23–37. 10.1007/s00429-019-01945-2. 10.1016/B978-0-444-64196-0.00002-9 31731913

[19] Petrosini L, Cutuli D, Picerni E, Laricchiuta D. Viewing the Personality Traits Through a Cerebellar Lens: a Focus on the Constructs of Novelty Seeking, Harm Avoidance, and Alexithymia. Cerebellum. 2017;16:178–90. 10.1007/s12311-015-0754-9. 10.1007/s12311-015-0754-9 26739351

[20] Van Overwalle F, Manto M, Cattaneo Z, Clausi S, Ferrari C, Gabrieli JDE et al. Consensus Paper: Cerebellum and Social Cognition. Cerebellum. 2020;19:833–86. 10.1007/s12311-020-01155-1. 10.1007/s12311-020-01155-1 PMC7588399 32632709

[21] Zhao Y, Li Q, Du J, He H, Liang P, Lu J et al. Exploring the Relationship between Gray and White Matter in Healthy Adults: A Hybrid Research of Cortical Reconstruction and Tractography. Biomed Res Int. 2021;2021. 10.1155/2021/6628506. 10.1155/2021/6628506 PMC7979294 33778072

[22] Agosta F, Pievani M, Sala S, Geroldi C, Galluzzi S, Frisoni GB et al. White matter damage in Alzheimer disease and its relationship to gray matter atrophy. Radiology. 2011;258:853–63. 10.1148/radiol.10101284. 10.1148/radiol.10101284 21177393

[23] Sasamoto A, Miyata J, Kubota M, Hirao K, Kawada R, Fujimoto S et al. Global association between cortical thinning and white matter integrity reduction in schizophrenia. Schizophr Bull. 2014;40:420–7. 10.1093/schbul/sbt030. 10.1093/schbul/sbt030 PMC3932083 23461997

[24] Herlin B, Navarro V, Dupont S. The temporal pole: From anatomy to function—A literature appraisal. J Chem Neuroanat. 2021;113:101925. 10.1016/j.jchemneu.2021.101925. 10.1016/j.jchemneu.2021.101925 33582250

[25] Veinante P, Yalcin I, Barrot M. The amygdala between sensation and affect: a role in pain. J Mol Psychiatry. 2013;1:9. 10.1186/2049-9256-1-9. 10.1186/2049-9256-1-9 PMC4223879 25408902

[26] Xu E, Nguyen L, Hu R, Stavish CM, Leibenluft E, Linke JO. The uncinate fasciculus in individuals with and at risk for bipolar disorder: A meta-analysis. J Affect Disord. 2022;297:208–16. 10.1016/j.jad.2021.10.045. 10.1016/j.jad.2021.10.045 PMC8631233 34699854

[27] Xu EP, Nguyen L, Leibenluft E, Stange JP, Linke JO. A meta-analysis on the uncinate fasciculus in depression. Psychol Med. 2023;53:2721–31. 10.1017/S0033291723000107. 10.1017/S0033291723000107 PMC10235669 37051913

[28] Gamal-Eltrabily M, Martínez-Lorenzana G, González-Hernández A, Condés-Lara M. Cortical Modulation of Nociception. Neuroscience. 2021;458:256–70. 10.1016/j.neuroscience.2021.01.001. 10.1016/j.neuroscience.2021.01.001 33465410

[29] Lu C, Yang T, Zhao H, Zhang M, Meng F, Fu H et al. Insular Cortex is Critical for the Perception, Modulation, and Chronification of Pain. Neurosci Bull. 2016;32:191–201. 10.1007/s12264-016-0016-y. 10.1007/s12264-016-0016-y PMC5563738 26898298

[30] Kim W, Kim SK, Nabekura J. Functional and structural plasticity in the primary somatosensory cortex associated with chronic pain. J Neurochem. 2017;141:499–506. 10.1111/jnc.14012. 10.1111/jnc.14012 28278355

[31] Obata H. Analgesic mechanisms of antidepressants for neuropathic pain. Int J Mol Sci. 2017;18. 10.3390/ijms18112483. 10.3390/ijms18112483 PMC5713449 29160850

[32] Wolfe F, Clauw DJ, Fitzcharles MA, Goldenberg DL, Katz RS, Mease P et al. The American College of Rheumatology preliminary diagnostic criteria for fibromyalgia and measurement of symptom severity. Arthritis Care Res. 2010;62:600–10. 10.1002/acr.20140. 10.1002/acr.20140 20461783

[33] Gupta A, Mayer EA, Fling C, Labus JS, Naliboff BD, Hong J-Y et al. Sex-based differences in brain alterations across chronic pain conditions. J Neurosci Res. 2017;95:604–16. 10.1002/jnr.23856. 10.1002/jnr.23856 PMC5120652 27870423

[34] Ceko M, Bushnell MC, Fitzcharles M, Schweinhardt P. Fibromyalgia interacts with age to change the brain. NeuroImage Clin. 2013;3:249–60. 10.1016/j.nicl.2013.08.015. 10.1016/j.nicl.2013.08.015 PMC3814958 24273710

[35] Radloff LS, The CES-D, Scale. A Self-Report Depression Scale for Research in the General Population. Appl Psychol Meas. 1977;1:385–401. 10.1177/014662167700100306.

[36] Shima S (1985). [New Self-Rating scales for Depression] Atarashii Yokuutsuseijikohyokasyakudo Ni tsuite (in Japanese). Seishin Igaku (Clinical Psychiatry).

[37] Shimizu H, Imae K (1981). [Development of the Japanese version of state-trait anxiety inventory] STATE-TRAIT ANXIETY INVENTORY no nihongoban (daigakuseiyo) no sakusei (in Japanese). Kyoiku Shinrigaku Kenkyu (Japanese J Educ Psychol).

[38] Spielberger CD, Gorsuch RL, Lushene R, Vagg PR, Jacobs GA. Manual for the state-trait anxiety inventory. Consult Psychol Press; 1983.

[39] Hatta H, Higashi A, Yashiro H, Ozawa K, Hayashi K, Kiyota K (1998). [Validation of the hospital anxiety and Depression Scale] Hospital anxiety and Depression Scale Nihongoban no shinraisei to datosei no kento josei wo taisyo to shita seiseki (in Japanese). Shinshin Igaku (Japanese J Psychosom Med.

[40] Zigmond AS, Snaith RP. The Hospital Anxiety and Depression Scale. Acta Psychiatr Scand. 1983;67:361–70. 10.1111/j.1600-0447.1983.tb09716.x. 10.1111/j.1600-0447.1983.tb09716.x 6880820

[41] Matsuoka H, Sakano Y (2007). [Assessment of Cognitive Aspect of Pain: development, reliability, and validation of Japanese Version of Pain Catastrophizing Scale] Itami no Ninchimen no hyoka: Pain Catastrophizing Scale Nihongoban no sakusei to shinraisei oyobi datosei no kento (in Japanese). Shinshin Igaku (Japanese J Psychosom Med).

[42] Sullivan MJL, Bishop SR, Pivik J. The Pain Catastrophizing Scale: Development and validation. Psychol Assess. 1995;7:524–32. 10.1037/1040-3590.7.4.524.

[43] Ashburner J. A fast diffeomorphic image registration algorithm. Neuroimage. 2007;38:95–113. 10.1016/j.neuroimage.2007.07.007. 10.1016/j.neuroimage.2007.07.007 17761438

[44] SERGENT J, OHTA S. MACDONALD B. Functional Neuroanatomy of Face and Object Processing. Brain. 1992;115:15–36. 10.1093/brain/115.1.15. 10.1093/brain/115.1.15 1559150

[45] Pobric G, Jefferies E, Lambon Ralph MA. Amodal semantic representations depend on both anterior temporal lobes: Evidence from repetitive transcranial magnetic stimulation. Neuropsychologia. 2010;48:1336–42. 10.1016/j.neuropsychologia.2009.12.036. 10.1016/j.neuropsychologia.2009.12.036 20038436

[46] Maguire EA, Mummery CJ. Differential modulation of a common memory retrieval network revealed by positron emission tomography. Hippocampus. 1999;9:54–61. 10.1002/(SICI)1098-1063(1999)9:1%3C54::AID-HIPO6%3E3.0.CO;2-O. 10.1002/(SICI)1098-1063(1999)9:1<54::AID-HIPO6>3.0.CO;2-O 10088900

[47] Reniers RLEP, Völlm BA, Elliott R, Corcoran R, Empathy. ToM, and self-other differentiation: An fMRI study of internal states. Soc Neurosci. 2014;9:50–62. 10.1080/17470919.2013.861360. 10.1080/17470919.2013.861360 24294841

[48] Cun L, Wang Y, Zhang S, Wei D, Qiu J. The contribution of regional gray/white matter volume in preclinical depression assessed by the Automatic Thoughts Questionnaire: A voxel-based morphometry study. Neuroreport. 2014;25:1030–7. 10.1097/WNR.0000000000000222. 10.1097/WNR.0000000000000222 24999908

[49] Liu Y, Zhang N, Bao G, Huang Y, Ji B, Wu Y et al. Predictors of depressive symptoms in college students: A systematic review and meta-analysis of cohort studies. J Affect Disord. 2019;244:196–208. 10.1016/j.jad.2018.10.084. 10.1016/j.jad.2018.10.084 30352363

[50] Flouri E, Panourgia C. Negative automatic thoughts and emotional and behavioural problems in adolescence. Child Adolesc Ment Health. 2014;19:46–51. 10.1111/camh.12004. 10.1111/camh.12004 32878364

[51] Zhao SR, Ni XM, Zhang XA, Tian H. Effect of cognitive behavior therapy combined with exercise intervention on the cognitive bias and coping styles of diarrheapredominant irritable bowel syndrome patients. World J Clin Cases. 2019;7:3446–62. 10.12998/wjcc.v7.i21.3446. 10.12998/wjcc.v7.i21.3446 PMC6854400 31750328

[52] Torelli P, Abrignani G, Castellini P, Lambru G, Manzoni GC. Human psyche and headache: Tension-type headache. Neurol Sci. 2008;29:93–5. 10.1007/s10072-008-0896-3. 10.1007/s10072-008-0896-3 18545906

[53] Ingram RE, Atkinson JH, Slater MA, Saccuzzo DP, Garfin SR. Negative and positive cognition in depressed and nondepressed chronic-pain patients. Heal Psychol. 1990;9:300–14. 10.1037/0278-6133.9.3.300. 10.1037//0278-6133.9.3.300 2140322

[54] Yang Z, Jackson T, Huang C. Neural activation during anticipation of near pain-threshold stimulation among the pain-fearful. Front Neurosci. 2016;10:1–9. 10.3389/fnins.2016.00342. 10.3389/fnins.2016.00342 PMC4951481 27489536

[55] Moulton EA, Becerra L, Maleki N, Pendse G, Tully S, Hargreaves R et al. Painful heat reveals hyperexcitability of the temporal pole in interictal and ictal migraine states. Cereb Cortex. 2011;21:435–48. 10.1093/cercor/bhq109. 10.1093/cercor/bhq109 PMC3020583 20562317

[56] Kuchinad A, Schweinhardt P, Seminowicz DA, Wood PB, Chizh BA, Bushnell MC. Accelerated Brain Gray Matter Loss in Fibromyalgia Patients: Premature Aging of the Brain? J Neurosci. 2007;27:4004–7. 10.1523/JNEUROSCI.0098-07.2007. 10.1523/JNEUROSCI.0098-07.2007 PMC6672521 17428976

[57] Schmidt-Wilcke T, Luerding R, Weigand T, Jürgens T, Schuierer G, Leinisch E et al. Striatal grey matter increase in patients suffering from fibromyalgia – A voxel-based morphometry study. Pain. 2007;132:S109–16. 10.1016/j.pain.2007.05.010. 10.1016/j.pain.2007.05.010 17587497

[58] Burgmer M, Gaubitz M, Konrad C, Wrenger M, Hilgart S, Heuft G et al. Decreased Gray Matter Volumes in the Cingulo-Frontal Cortex and the Amygdala in Patients With Fibromyalgia. Psychosom Med. 2009;71:566–73. 10.1097/PSY.0b013e3181a32da0. 10.1097/PSY.0b013e3181a32da0 19414621

[59] Hsu MC, Harris RE, Sundgren PC, Welsh RC, Fernandes CR, Clauw DJ et al. No consistent difference in gray matter volume between individuals with fibromyalgia and age-matched healthy subjects when controlling for affective disorder. Pain. 2009;143:262–7. 10.1016/j.pain.2009.03.017. 10.1016/j.pain.2009.03.017 PMC2719961 19375224

[60] Robinson ME, Craggs JG, Price DD, Perlstein WM, Staud R. Gray Matter Volumes of Pain-Related Brain Areas Are Decreased in Fibromyalgia Syndrome. J Pain. 2011;12:436–43. 10.1016/j.jpain.2010.10.003. 10.1016/j.jpain.2010.10.003 PMC3070837 21146463

[61] Fallon N, Alghamdi J, Chiu Y, Sluming V, Nurmikko T, Stancak A. Structural alterations in brainstem of fibromyalgia syndrome patients correlate with sensitivity to mechanical pressure. NeuroImage Clin. 2013;3:163–70. 10.1016/j.nicl.2013.07.011. 10.1016/j.nicl.2013.07.011 PMC3791285 24179860

[62] Wolfe F, Smythe HA, Yunus MB, Bennett RM, Bombardier C, Goldenberg DL (1990). The American College of Rheumatology 1990 Criteria for the classification of Fibromyalgia. Report of the Multicenter Criteria Committee. Arthritis Rheum.

[63] Babaev O, Piletti Chatain C, Krueger-Burg D. Inhibition in the amygdala anxiety circuitry. Exp Mol Med. 2018;50. 10.1038/s12276-018-0063-8. 10.1038/s12276-018-0063-8 PMC5938054 29628509

[64] Chen M-H, Sun C-K, Lin I-M, Suen M-W, Sue Y-R, Chen I-L et al. Size Reduction of the Right Amygdala in Chronic Pain Patients with Emotional Stress: A Systematic Review and Meta-Analysis. Pain Med. 2023;24:556–65. 10.1093/pm/pnac162. 10.1093/pm/pnac162 36308460

[65] Baur V, Hänggi J, Jäncke L. Volumetric associations between uncinate fasciculus, amygdala, and trait anxiety. BMC Neurosci. 2012;13:4. 10.1186/1471-2202-13-4. 10.1186/1471-2202-13-4 PMC3398321 22217209

[66] Blackmon K, Barr WB, Carlson C, Devinsky O, DuBois J, Pogash D et al. Structural evidence for involvement of a left amygdala-orbitofrontal network in subclinical anxiety. Psychiatry Res Neuroimaging. 2011;194:296–303. 10.1016/j.pscychresns.2011.05.007. 10.1016/j.pscychresns.2011.05.007 PMC3544472 21803551

[67] Hayano F, Nakamura M, Asami T, Uehara K, Yoshida T, Roppongi T et al. Smaller amygdala is associated with anxiety in patients with panic disorder. Psychiatry Clin Neurosci. 2009;63:266–76. 10.1111/j.1440-1819.2009.01960.x. 10.1111/j.1440-1819.2009.01960.x 19566756

[68] Soriano-Mas C, Hernndez-Ribas R, Pujol J, Urretavizcaya M, Deus J, Harrison BJ et al. Cross-sectional and longitudinal assessment of structural brain alterations in melancholic depression. Biol Psychiatry. 2011;69:318–25. 10.1016/j.biopsych.2010.07.029. 10.1016/j.biopsych.2010.07.029 20875637

[69] Venkatraman A, Edlow BL, Immordino-Yang MH. The Brainstem in Emotion: A Review. Front Neuroanat. 2017;11:1–12. 10.3389/fnana.2017.00015. 10.3389/fnana.2017.00015 PMC5343067 28337130

[70] Abe O, Yamasue H, Kasai K, Yamada H, Aoki S, Inoue H et al. Voxel-based analyses of gray/white matter volume and diffusion tensor data in major depression. Psychiatry Res - Neuroimaging. 2010;181:64–70. 10.1016/j.pscychresns.2009.07.007. 10.1016/j.pscychresns.2009.07.007 19959342

[71] Luo L, Wu K, Lu Y, Gao S, Kong X, Lu F et al. Increased Functional Connectivity Between Medulla and Inferior Parietal Cortex in Medication-Free Major Depressive Disorder. Front Neurosci. 2018;12:1–7. 10.3389/fnins.2018.00926. 10.3389/fnins.2018.00926 PMC6295569 30618555

[72] Suárez-Pereira I, Llorca-Torralba M, Bravo L, Camarena-Delgado C, Soriano-Mas C, Berrocoso E. The Role of the Locus Coeruleus in Pain and Associated Stress-Related Disorders. Biol Psychiatry. 2022;91:786–97. 10.1016/j.biopsych.2021.11.023. 10.1016/j.biopsych.2021.11.023 35164940

[73] Lutz J, Jäger L, De Quervain D, Krauseneck T, Padberg F, Wichnalek M et al. White and gray matter abnormalities in the brain of patients with fibromyalgia: A diffusion-tensor and volumetric imaging study. Arthritis Rheum. 2008;58:3960–9. 10.1002/art.24070. 10.1002/art.24070 19035484

[74] Irimia A, Labus JS, Torgerson CM, Van Horn JD, Mayer EA. Altered viscerotopic cortical innervation in patients with irritable bowel syndrome. Neurogastroenterol Motil. 2015;27:1075–81. 10.1111/nmo.12586. 10.1111/nmo.12586 PMC4520752 25952540

[75] Yoon EJ, Kim YK, Shin HI, Lee Y, Kim SE. Cortical and white matter alterations in patients with neuropathic pain after spinal cord injury. Brain Res. 2013;1540:64–73. 10.1016/j.brainres.2013.10.007. 10.1016/j.brainres.2013.10.007 24125807

